# Development and Evaluation of Physiologically Based Pharmacokinetic Drug–Disease Models for Predicting Rifampicin Exposure in Tuberculosis and Cirrhosis Populations

**DOI:** 10.3390/pharmaceutics11110578

**Published:** 2019-11-05

**Authors:** Muhammad F. Rasool, Sundus Khalid, Abdul Majeed, Hamid Saeed, Imran Imran, Mohamed Mohany, Salim S. Al-Rejaie, Faleh Alqahtani

**Affiliations:** 1 Department of Pharmacy Practice, Faculty of Pharmacy, Bahauddin Zakariya University, Multan 60800, Pakistan; abdulmajeed@bzu.edu.pk; 2 Department of Pharmaceutics, Faculty of Pharmacy, Bahauddin Zakariya University, Multan 60800, Pakistan; sunduskhalid.sk@gmail.com; 3 Section of Pharmaceutics, University College of Pharmacy, Allama Iqbal Campus, University of the Punjab, Lahore 54000, Pakistan; hamid.pharmacy@pu.edu.pk; 4 Department of Pharmacology, Faculty of Pharmacy, Bahauddin Zakariya University, Multan 60800, Pakistan; imran.ch@bzu.edu.pk; 5 Department of Pharmacology and Toxicology, College of Pharmacy, King Saud University, Riyadh 11451, Saudi Arabia; mmohany@ksu.edu.sa (M.M.); rejaie@ksu.edu.sa (S.S.A.-R.)

**Keywords:** PBPK, Simcyp^®^, drug–disease model, Rifampicin, pharmacokinetics, tuberculosis, cirrhosis, clearance

## Abstract

The physiologically based pharmacokinetic (PBPK) approach facilitates the construction of novel drug–disease models by allowing incorporation of relevant pathophysiological changes. The aim of the present work was to explore and identify the differences in rifampicin pharmacokinetics (PK) after the application of its single dose in healthy and diseased populations by using PBPK drug–disease models. The Simcyp^®^ simulator was used as a platform for modeling and simulation. The model development process was initiated by predicting rifampicin PK in healthy population after intravenous (i.v) and oral administration. Subsequent to successful evaluation in healthy population, the pathophysiological changes in tuberculosis and cirrhosis population were incorporated into the developed model for predicting rifampicin PK in these populations. The model evaluation was performed by using visual predictive checks and the comparison of mean observed/predicted ratios (ratio_(Obs/pred)_) of the PK parameters. The predicted PK parameters in the healthy population were in adequate harmony with the reported clinical data. The incorporation of pathophysiological changes in albumin concentration in the tuberculosis population revealed improved prediction of clearance. The developed PBPK drug–disease models have efficiently described rifampicin PK in tuberculosis and cirrhosis populations after administering single drug dose, as the ratio_(Obs/pred)_ for all the PK parameters were within a two-fold error range. The mechanistic nature of the developed PBPK models may facilitate their extension to other diseases and drugs.

## 1. Introduction

Physiologically based pharmacokinetic (PBPK) approach has gained value in recent years as it provides novel opportunities for the prediction of systemic drug concentrations in both healthy and diseased populations [1,2,3]. The PBPK models have been utilized to predict inter-individual variability associated with the absorption, distribution, metabolism and excretion (ADME) of administered drugs [4,5,6]. Moreover, by providing an opportunity to incorporate pathophysiological changes occurring in a disease, the PBPK approach facilitates the construction of drug–disease models [7,8,9,10,11]. Once a PBPK drug–disease model is developed and evaluated, due to its mechanistic nature, it can easily be extended to other populations and drugs [12,13]. In comparison with the classical top-down modeling approach, the PBPK models have the capacity to allow incorporation of disease-specific changes that can facilitate in construction of drug–disease models [14]. Therefore, the PBPK approach can be very useful in predicting ADME of administered drugs in clinically important highly prevalent diseases (e.g., Tuberculosis).

Tuberculosis is a contagious yet curable disease affecting one-third of the world’s population [15]. Administration of sub-therapeutic doses in tuberculosis may contribute towards the development of drug resistance [16]. In tuberculosis patients, the inter-individual variation in drug response may be linked with the reported changes in plasma albumin concentration [17,18]. These alterations in plasma albumin levels may have an impact on ADME of low hepatic clearance drugs [19]. 

Rifampicin is the major drug used in treatment of tuberculosis and is metabolized mainly in the liver by various cytochrome-P450 (CYP) enzymes [20]. Rifampicin is a drug with low hepatic clearance and has a bioavailability of 93–95% [21,22]. Its volume of distribution is 0.33–0.53 L/kg and is highly bound to plasma proteins with a short half-life [23,24,25]. The changes in albumin concentrations occurring in tuberculosis may affect ADME of low hepatic clearance drugs like rifampicin. Therefore, if a PBPK drug–disease model is developed that can incorporate changes in the plasma protein binding occurring in tuberculosis patients, it can be used to predict the impact of these changes. Furthermore, rifampicin is in clinical use for the last few decades with plethora of clinical pharmacokinetic (PK) data in the literature [15,26,27,28], which makes it more befitting model drug for the development and evaluation of rifampicin-tuberculosis drug–disease model. Additionally, the developed PBPK model can also be extended to other chronic conditions such as liver cirrhosis where the pathophysiological changes in intrinsic clearance (*CL_int_*) of hepatic enzymes and plasma protein binding can have a profound effect on the PK of rifampicin [17,29,30].

The previously published PBPK models for rifampicin were focused on prediction of drug–drug interactions (DDIs) by incorporating CYP3A4 induction after administration of its multiple doses [25,31]. The primary focus of the presented work was to explore and identify the factors that contribute towards differences in rifampicin PK after application of its single dose in healthy and disease populations (tuberculosis and cirrhosis). The main objective of the study was to develop PBPK drug–disease models that incorporate the relevant pathophysiological changes occurring in tuberculosis and cirrhosis populations by using rifampicin (a low hepatic clearance drug) as a model drug. 

## 2. Materials and Methods 

### 2.1. Modeling Software and Strategy 

The population-based PBPK simulator Simcyp^®^ version 16.1 (Simcyp Ltd., Sheffield, UK) was used as a platform for modeling and simulation of ADME of the selected drug. The software assimilates both in vitro and in vivo ADME data along with the physicochemical properties of the drug for evaluating the drug’s exposure in both healthy and diseased populations [32]. 

A systematic model building approach was used for PBPK model development, the process was initiated by extracting drug-specific parameters and pharmacokinetic profiles from the published literature [33]. In order to select and finalize the model input parameters that govern drug distribution and clearance, the simulations were performed in healthy population after intravenous (i.v) application. The model input parameters like fraction unbound (*f*_u_) and blood to plasma ratio (B:P) were optimized at this stage by performing sensitivity analysis and manual optimization after comparing reported and predicted PK parameters. Subsequent to evaluation of the i.v predictions with the available clinical data, absorption related parameters, like human jejunum permeability (P_eff,man_) was optimized on the basis of comparison of reported and predicted values of time to reach maximum systemic drug concentration (T_max_) and was incorporated into the model for predicting drug PK after oral application.

**Figure 1:**
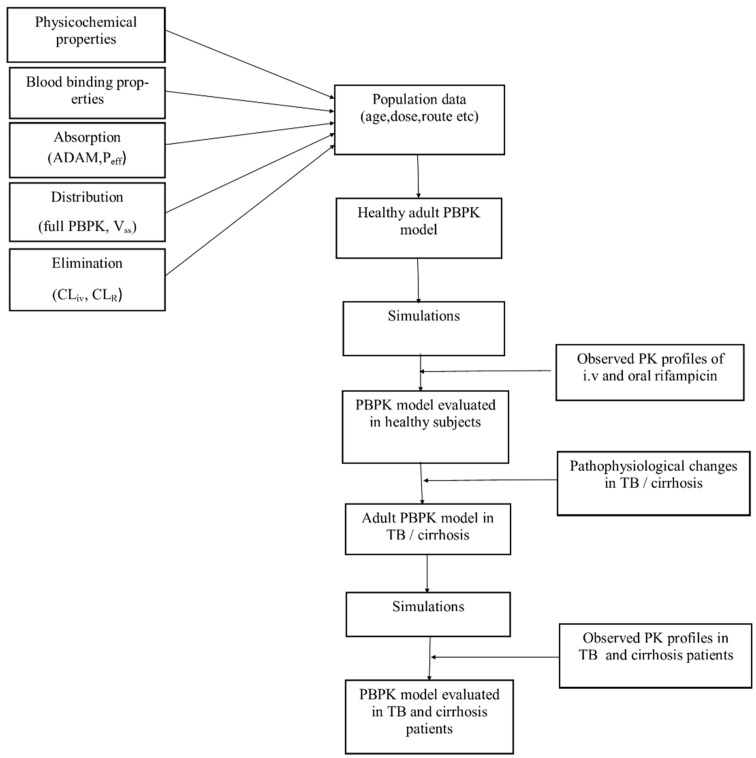
Systematic diagram for the development and evaluation of rifampicin PBPK models in healthy and diseased patients. PBPK; Physiologically based pharmacokinetic, ADAM: advanced dissolution, absorption and metabolism, P_eff_: human jejunum permeability, V_ss_: volume of distribution at steady-state, CL_iv_: intravenous clearance, CL_R_: renal clearance, PK: pharmacokinetic, i.v: intravenous, TB: tuberculosis.

The oral predictions were then evaluated with the reported clinical data. After successful evaluation of the developed model in the healthy population, the plasma albumin changes occurring in tuberculosis and the pathophysiological changes in organ blood flows, hepatic enzyme abundance and liver volume, etc., occurring in cirrhosis were incorporated into the developed model for predicting drug ADME in these disease populations. The systematic diagram for the developed PBPK models can be seen as in Figure 1.

### 2.2. Model Structure

The model parameterization was initiated by thoroughly reviewing the in vitro and in vivo ADME data for rifampicin in the published literature. The final rifampicin specific input parameters for the developed PBPK model are given in Table 1. The description of various model components is given below.

#### 2.2.1. Physicochemical Properties

Rifampicin (C_45_H_58_N_4_O_12_) is an ampholytic compound, having a molecular weight of 822.97 g/mol with pK_a_ value of 1.7, 7.9 and logP value of 2.7 [24,34]. Further details for physicochemical parameters can be seen in Table 1.

#### 2.2.2. Absorption 

The advanced dissolution, absorption and metabolism (ADAM) model was used for the prediction of oral drug absorption [35]. The reported value of P_eff_: 2.15 × 10^−4^ cm/s [23] was associated with delayed prediction of time to reach maximum plasma concentration (T_max_). This value was optimized to 2.4 × 10^−4^ cm/s by performing sensitivity analysis and manual optimization after comparing reported and predicted values of T_max_. 

#### 2.2.3. Distribution

A whole-body full PBPK model was used for the prediction of drug distribution. The Rodger and Rowland method (method-2 within the Simcyp^®^) was used for predicting volume of distribution at steady-state (V_ss_) [36]. The predicted V_ss_ value of 0.48 L/kg was comparable to the reported range of 0.33–0.53 L/kg in the literature [23,24,25]. 

#### 2.2.4. Elimination

In the developed model the hepatic clearance (CL_H_) was assigned as intravenous clearance (CL_iv_). The CL_iv_ and renal clearance (CL_R_) values of 7 L/h [24] and 1.5 L/h [25] respectively were used for predicting rifampicin clearance. 

### 2.3. Population Specific (System) Data 

The demographic, anatomic and physiologic parameters that were used in the creation of virtual populations (healthy and disease) were based on Simcyp population libraries. 

#### 2.3.1. Disease-Specific Pathophysiological Changes

##### Tuberculosis

It has been reported that patients with tuberculosis have lower plasma albumin levels [37]. This decrease in plasma albumin concentration can lead to alterations in the free fraction of administered drugs in tuberculosis patients [37]. Since rifampicin is a drug with low hepatic clearance and is mainly bound to plasma albumin, therefore its ADME may alter with changes in plasma albumin concentration [19]. In tuberculosis, the reported reduction in plasma albumin levels ranges between 30–39 g/L [17]. Based on the comparison between observed vs. predicted PK profiles, the plasma albumin value in the developed PBPK model was reduced to 38 g/L in tuberculosis population for prediction of rifampicin ADME. The tuberculosis patients generally weigh less, and this was accounted for during creation of virtual tuberculosis population. The weight range of the simulated tuberculosis population was comparable with that of the reported clinical study. 

##### Liver Cirrhosis

Liver cirrhosis is associated with a wide range of pathophysiological changes that can influence the PK of administered drugs. This includes alterations in: CL_int_ of metabolic enzymes, hepatic blood flow, renal blood flow, gastric emptying, liver volume and albumin concentrations [9,38]. The severity of liver disease is usually assessed by Child–Pugh (CP) score [9]. By using CP score we can categorize liver cirrhosis patients into three categories i.e., CP-A (mild impairment), CP-B (moderate impairment) and CP-C (severe impairment) [38]. The liver cirrhosis population is stratified into CP classes (A–C) within Simcyp^®^ [38]. 

### 2.4. Ethics

No ethical approval was required for this study as the clinical PK data sets used for model evaluation in healthy and disease patients were sourced from published studies (Table 2 and Table 3). The reported mean systemic rifampicin concentration vs. time profiles were scanned by using GetData Graph Digitizer (version 2.26) [39].

### 2.5. Pharmacokinetic Data

Extensive literature searches were performed using various online search engines: PubMed and Google Scholar to screen for rifampicin related PK studies. Initial screening of PK studies was based on the presence of rifampicin plasma concentration vs. time profiles in the literature. The final selection of reported rifampicin PK studies was based on the presence of clear information on administered dose, disease state, fasting/fed state, the proportion of females and age. Finally, 22 clinical studies and 36 mean systemic rifampicin concentration vs. time profiles were included in the model development and evaluation process, among which 15 studies with 26 profiles (i.v = 2 and oral = 24) and 358 healthy individuals were included. Five studies were in tuberculosis patients with 6 profiles and 122 individuals and 1 in cirrhosis (CP-A) with 4 profiles and 28 individuals were used. The data used for rifampicin model development and evaluation in healthy and disease populations are given in Table 2 and Table 3. 

### 2.6. Model Evaluation

Simulations were executed by selecting a population of 100 individuals as there was no significant difference in prediction results after using a higher number of virtual individuals (200 or 300). Therefore, as seen in other published studies [54,55,56,57,58,59] a population of 100 virtual individuals with same age range, dosing, route of administration, female proportion, and fed/fast state as mentioned in the reference study was used in all the simulations. The model evaluation was performed by using visual predictive checks and comparison of predicted and observed values of PK parameters. The PK parameters, area under the plasma concentration vs. time curve from time zero to infinity (AUC_0–∞_), maximum plasma concentration (C_max_) and clearance (CL) were used for comparison between observed and predicted data. A non-compartmental analysis (NCA) was performed on observed and predicted plasma concentration vs. time profiles using PK SOLVER program [60]. Moreover, mean observed/predicted ratios for AUC_0–∞_, C_max_ and CL along with their 95% confidence intervals (CI), average fold error (AFE) and root mean square error (RMSE) were used for model evaluation. The values of ratio_(Obs/pred)_, AFE and RMSE were calculated separately for each population by using Equations (1)–(3). The fold error was calculated by dividing the observed value of the PK parameter with its predicted value. A two-fold error range was used as a reference for evaluation of ratio_(Obs/pred)_ for PK parameters. In order to fall within the two-fold error range, the ratio_(Obs/pred)_ of the PK parameters should be within 0.5–2-fold range [33,38,61,62,63]. Furthermore, the mean observed and predicted values for the PK parameters along with their range were also used for comparing results. The mean observed and predicted values of PK parameters refer to the mean of all the mean observed and predicted rifampicin profiles that were used for model evaluation. Since, there were only two iv studies that were used for model evaluation in healthy population, the ratio_(Obs/pred)_ for PK parameters was reported as mean with range instead of mean with 95% CI. Lastly, the goodness of fit plots were used for the identification of systematic errors in the model predictions. 

Mean observed/predicted ratio

(1) $$ratio_{\left(\frac{obs}{pred}\right)}=\frac{observed\;value\;of\;PK\;parameter}{predicted\;value\;of\;PK\;parameter}$$

Average fold error

(2) $$AFE=\;10^{\frac{\sum \log\left(fold\;error\right)}{N}}$$

Root mean square error

(3) $$RMSE=\;\sqrt{\frac{\sum_{1}^{N}{\left(observed\;value\;of\;PK\;parameter\;-\;predicted\;value\;of\;PK\;parameter\;\right)}^{2}}{N}}$$

### 2.7. Simulations in Different Clinical Scenarios

The developed model was also used to predict rifampicin PK in different clinical scenarios, where no clinical data was available as there was only one clinical study in cirrhosis patients with Child–Pugh-A class and simulations were performed with same administered oral doses in Child–Pugh B and C populations so that rifampicin exposure can be compared between healthy and disease populations. Similarly, rifampicin exposure after i.v application was also predicted to show differences in AUC between healthy and disease populations.

## 3. Results

### 3.1. Healthy Population

**Figure 2:**
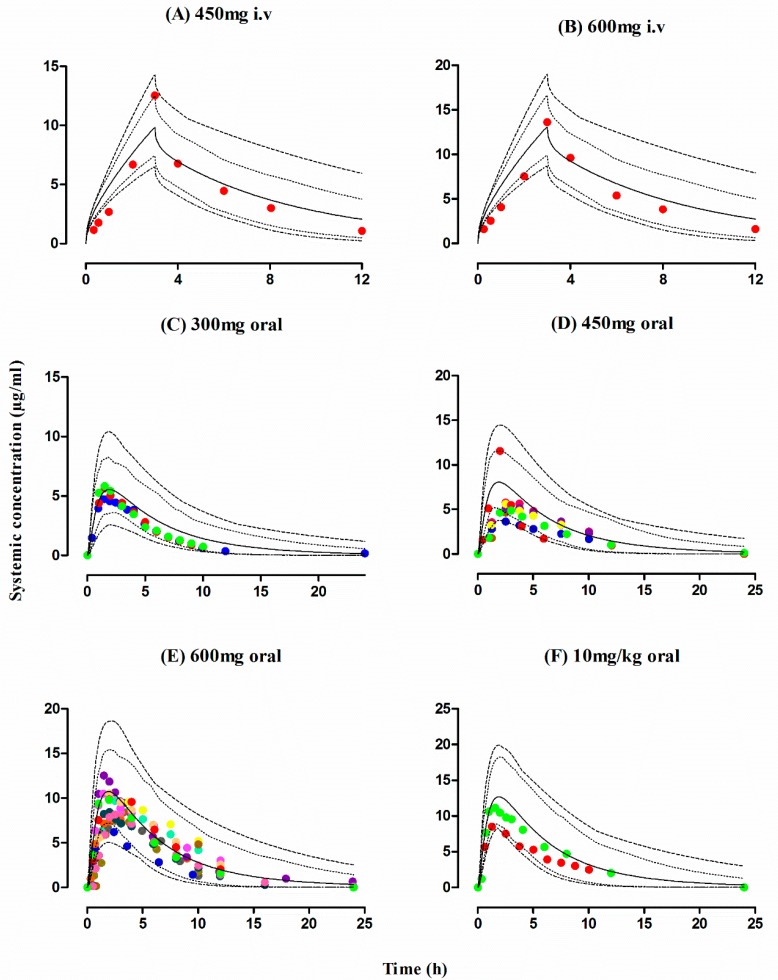
Observed and predicted plasma concentration-time profiles of rifampicin in healthy adults after iv and oral administration. Healthy individual after i.v administration: (**A**) 450 mg, (**B**) 600 mg [40]. Healthy individual after oral administration: (**C**) 300 mg [41,49], (**D**) 600 mg [26,27,28,42,43,44,45,46], (**E**) 450 mg [37,45,48], (**F**) 10 mg/kg [15,47]. The observed data are shown as filled colored circles. The predicted results are shown as mean (solid line), maximum value and minimum value (dashed line) and the 5th–95th percentiles (dotted line).

**Figure 3:**
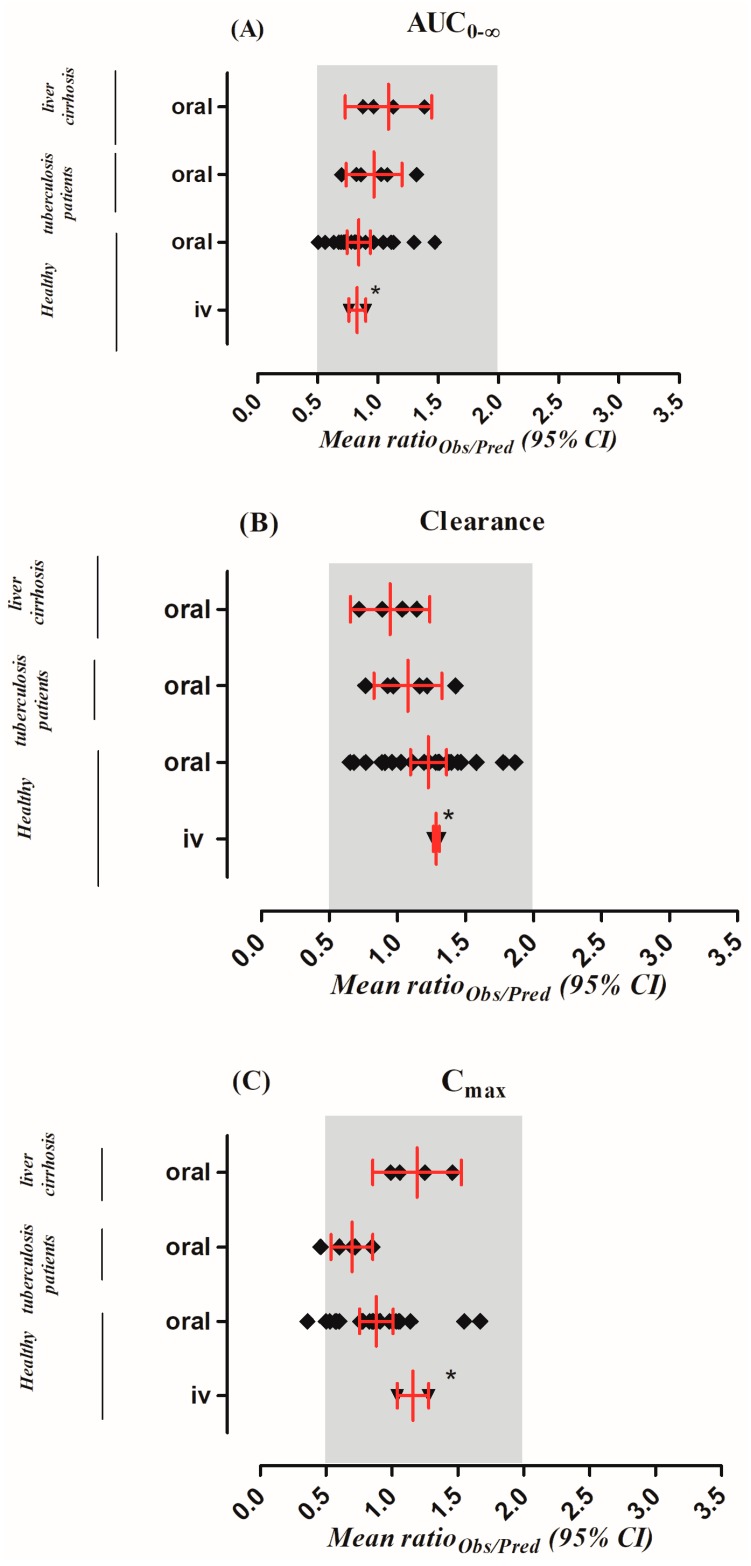
Comparison of observed and predicted pharmacokinetic parameters. The area under the curve from time 0 to the infinity (AUC_0–∞_) (**A**), drug clearance (CL) (**B**) and the maximum systemic concentration (C_max_) (**C**) in healthy, tuberculosis and cirrhosis populations. Results are represented as mean observed/predicted ratio [ratio_(Obs/Pred)_] values with 95% confidence interval. * value presented as mean with range.

**Figure 4:**
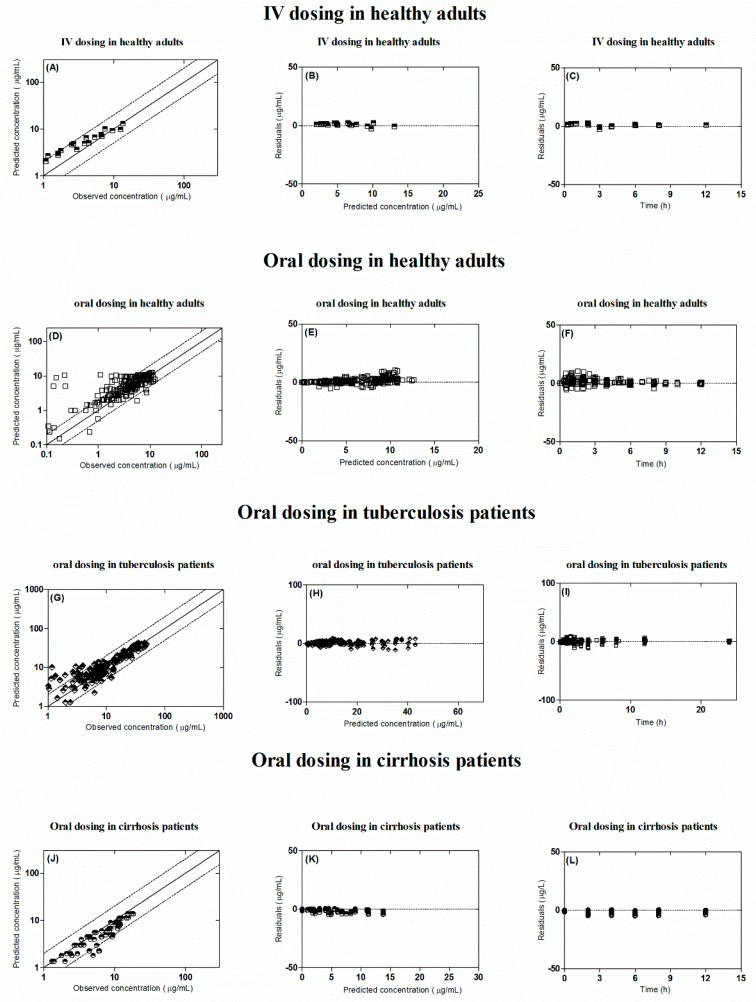
The goodness of fit plots in healthy and diseased population. (**A**,**D**,**G**,**J**) show predicted vs. observed systemic concentration plots. (**B**,**E**,**H**,**K**) show residuals vs. predicted systemic concentration plots. (**C**,**F**,**I**,**L)** show residuals vs. time plots. Intravenous application in healthy population: (**A**–**C**). Oral application in healthy population: (**D**–**F**). Oral application in tuberculosis patients: (**G**–**I**). Oral application in cirrhosis patients: (**J**–**L**). The square, diamonds and circles represent the observed and predicted systemic rifampicin concentrations. The solid line indicates line of identity; dash line indicates two-fold error range.

The observed and predicted PK profiles of rifampicin after i.v and oral administration in healthy individuals are shown in Figure 2. It is evident from the visual predictive checks that the model has captured the observed data effectively after i.v doses of 450–600 mg and oral doses of 300–600 mg, 10 mg/kg. Moreover, the mean AUC_0–∞_ ratio_(Obs/pred)_ after i.v and oral application were 0.82 (range: 0.76–0.89) and 0.84 (95% CI 0.74–0.94), respectively and the ratio_(Obs/pred)_ for C_max_ and CL were also within the allowed two-fold error range (Table 4 and Table 5, Figure 3). Additionally, residual plots demonstrated that there was no systematic error in model predictions (Figure 4A–F). The median observed and predicted plasma concentration-time profiles of rifampicin in healthy adults can be seen in supplementary Figure S1. 

### 3.2. Tuberculosis Patients

**Figure 5:**
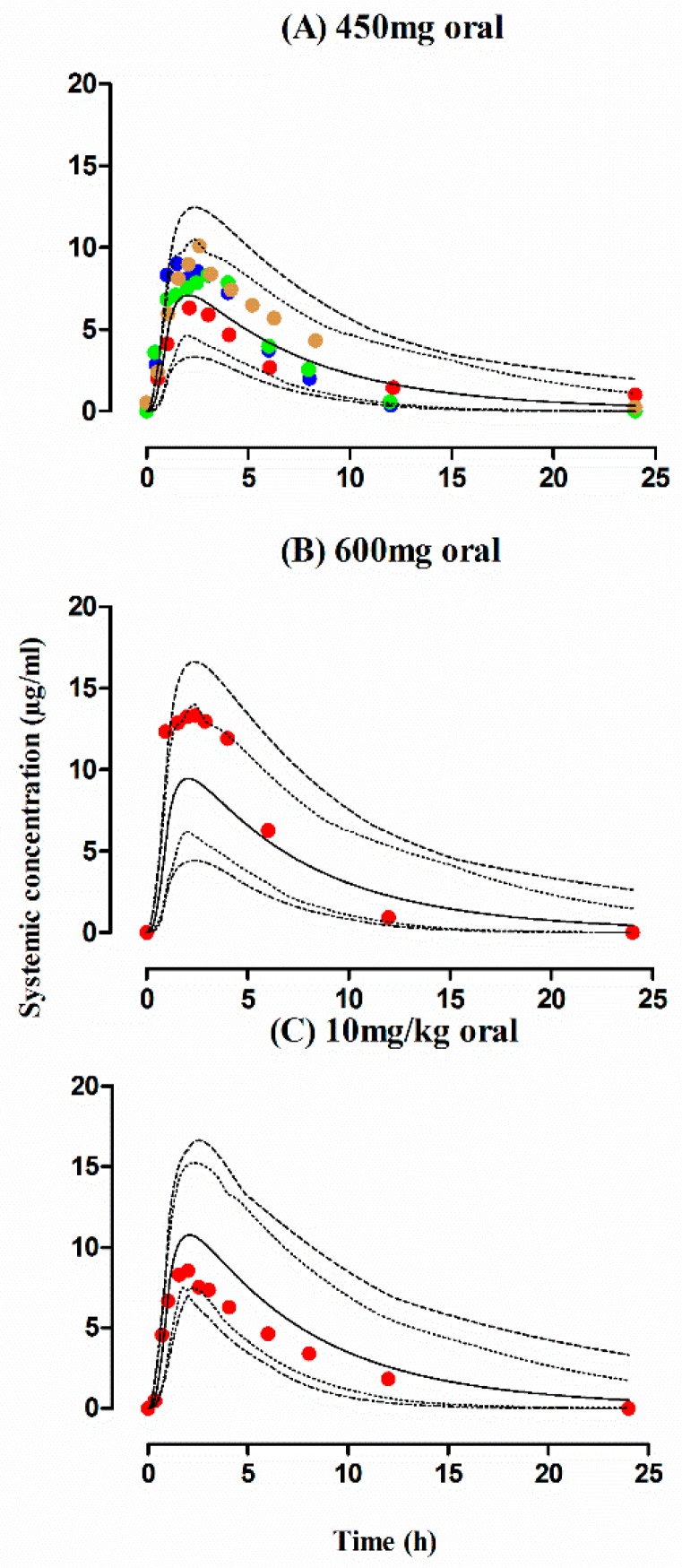
Observed and predicted plasma concentration-time profiles of rifampicin in tuberculosis patients after oral administration. Tuberculosis patients after oral administration (**A**) 450 mg/kg [37,50,51,52], (**B**) 600 mg [50], (**C**) 10 mg/kg [15]. The observed data are shown as filled colored circles. The predicted results are shown as mean (solid line), maximum value and minimum value (dashed line) and the 5th–95th percentiles (dotted line).

**Figure 6:**
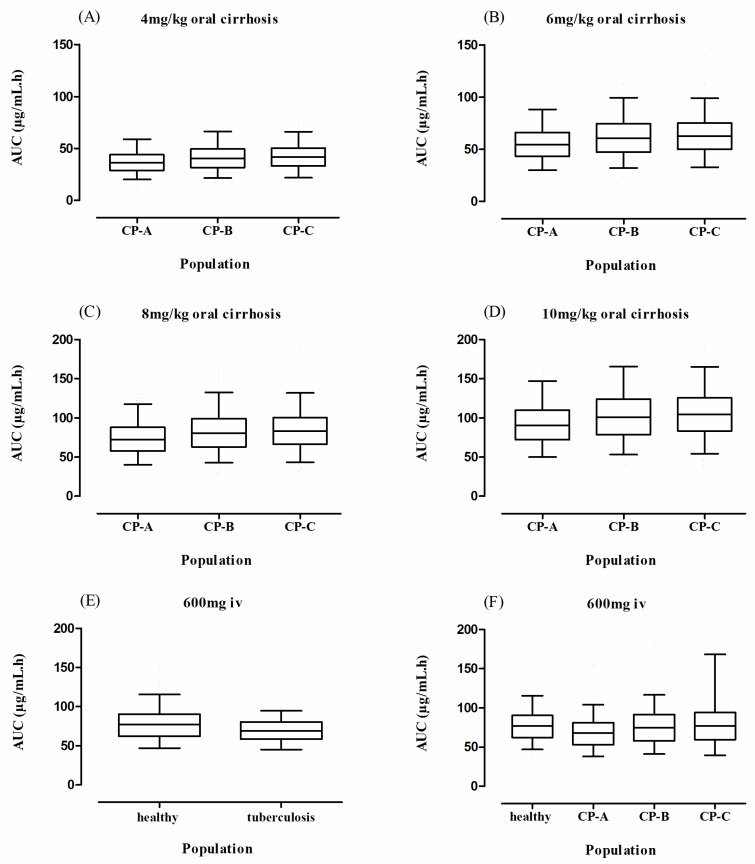
Box plots showing the mean area under the curve (AUC) along with 5th–95th percentiles confidence intervals (CI) for oral and i.v rifampicin in healthy, cirrhosis (**A**–**D**,**F**) and tuberculosis (**E**) populations. CP: Child–Pugh class.

The predicted outcomes of the model after oral administration of rifampicin in tuberculosis patients were in complete harmony with the observed data (Figure 5) and this was further confirmed by looking into the residual plots (Figure 4G–I). It can be seen from the systemic drug concentration vs. time plots that the model predictions have captured the observed after administering oral doses between 450–600 mg and 10 mg/kg. Furthermore, the AFE and RMSE for CL/F were 1.02 and 1.04 respectively. The ratio _(Obs/pred)_ for all the PK parameters were within the two-fold error range (Table 4 and Table 5, Figure 3). The model predictions showed that there was decrease in rifampicin AUC after application of 600 mg single dose of i.v rifampicin in tuberculosis patients as the mean AUC (µg/mL·h) reduced from 78.1 (range: 38.5–147.6) in healthy population to 70.6 (range: 37.6–122.3) in tuberculosis population (Figure 6E).

### 3.3. Liver Cirrhosis Patients

**Figure 7:**
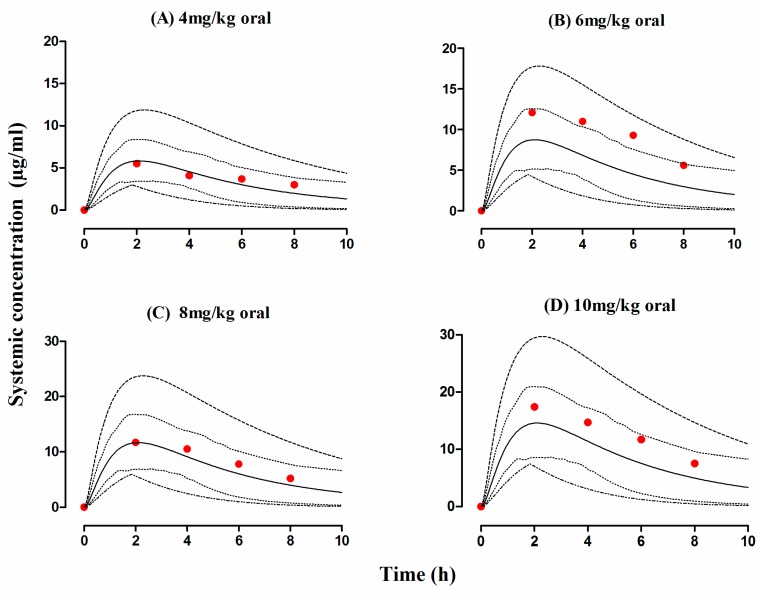
Observed and predicted plasma concentration-time profiles of rifampicin in cirrhosis patients after oral administration. Cirrhosis patients after oral administration (**A**) 4 mg/kg, (**B**) 6 mg/kg, (**C**) 8 mg/kg and (**D**) 10 mg/kg [53]. The observed data are shown as filled colored circles. The predicted results are shown as mean (solid line), maximum value and minimum value (dashed line) and the 5th–95th percentiles (dotted line).

The developed cirrhosis model effectively predicted rifampicin concentration time profiles after administering an oral dose of 4–10 mg/kg in cirrhosis patients. The visual predictive checks (Figure 7) and residual plots (Figure 4J–L) showed that these predictions were in complete agreement with the observed clinical data. Additionally, the AFE and RMSE values for CL/F (0.98 and 0.009 respectively) showed that the developed model has adequately captured rifampicin disposition in cirrhosis patients. The ratio_(Obs/pred) for_ AUC_0–∞_ and C_max_, were within the two-fold error range (Table 4 and Table 5, Figure 3). The simulated results showed that there was an increase in rifampicin AUC after oral application in cirrhosis populations (CP-A–C). A decrease in predicted AUC was seen in cirrhosis CP-A and B populations in comparison with healthy population after application of 600 mg i.v rifampicin (Figure 6A–D,F). 

## 4. Discussion

In the presented study, pathophysiological changes occurring in tuberculosis and cirrhosis were integrated into a whole-body PBPK model for the prediction of rifampicin exposure. The developed disease models were effective in predicting rifampicin PK in both tuberculosis and cirrhosis populations after single-dose application.

The developed model has captured the rifampicin disposition in a healthy population after i.v drug administration, as evident by an accord between the mean observed and predicted CL values of 8.0 L/h (range: 7.86–8.15) [40] and 6.2 L/h, respectively. Similarly, the mean observed and predicted CL/F values after oral rifampicin administration were 9.2 L/h (range: 5.82–13.7 L/h) [15,26,27,28,37,41,42,43,44,45,46,47,48,49] and 7.4 L/h (range: 7.02–9.60), respectively. Moreover, the AFE for the AUC_0–∞_ and C_max_ after i.v and oral administration was 0.78 and 0.80, 1.14 and 0.79, respectively, suggesting that the developed model has successfully captured the oral drug absorption process. Furthermore, the developed PBPK model has effectively predicted the drug bioavailability (F) in healthy population i.e., 84% (range 41–95%) which was comparable with the observed value of 93–95% [21].

It has been reported that the plasma albumin concentrations are altered in tuberculosis patients and these changes can potentially affect the ADME of low hepatic clearance drugs [17,19]. Since rifampicin is a drug with low hepatic clearance, thus its PK is susceptible to the changes in its free fraction [19,22]. The unbound fraction (*f*_u_) of rifampicin is increased in tuberculosis patients as the plasma albumin concentration declines, which in turn can increase the CL/F of rifampicin [64]. Conversely, in the present study, a slight reduction in rifampicin CL/F was seen. The mean observed and predicted rifampicin CL/F values were slightly reduced from 9.2 L/h (range: 5.82–13.7 L/h) L/h and 7.4 L/h (range: 7.022–9.60) in healthy population [15,26,27,28,37,41,42,43,44,45,46,47,48,49] to 7.0 L/h (range: 4.97–8.44) and 6.90 L/h (range: 6.5–7.25), respectively, in tuberculosis population [15,37,50,51,52]. The developed rifampicin-tuberculosis PBPK model has successfully captured this change in CL/F that is evident from the mean CL/F ratio_(Obs/pred)_ of 1.08 (95% CI 0.82–1.32) and RMSE of 1.04.

Theoretically, the increase in *f*_u_ of low hepatic clearance drugs like rifampicin in tuberculosis patients may lead to an increase in its CL/F, but in our study a decline in observed and reported rifampicin CL/F has been seen. This finding can be supported by the fact that the exposure of low hepatic clearance drugs that are metabolized primarily in liver is not dependent upon changes in their plasma protein binding [65]. Moreover, protein binding changes can have a significant impact on ADME of administered drug when a high non-hepatic clearance drug is administered intravenously, or a high hepatic clearance and a narrow therapeutic index drug is administered orally [65]. 

The administration of multiple doses of rifampicin in tuberculosis patients is associated with an increase in CL/F, as its value is increased from 4.5 L/h on day 1 to 6.8 L/h on day 14 [66]. This increase in rifampicin CL/F is associated with the induction of hepatic enzymes [67]. Since, rifampicin is hepatically metabolized by CYP-enzymes (1A2, 2C9, 2C19, 3A4 and 3A5) and it has the ability to induce these enzymes, that is why its CL/F is increased in tuberculosis patients receiving multiple doses of rifampicin [67,68]. Moreover, it has been seen that rifampicin CL/F is also increased in malnourished tuberculosis patients [69]. This increase in rifampicin CL/F in malnourished tuberculosis patients is directly linked with poor drug absorption along with low serum albumin concentrations and induction of hepatic enzymes after administration of multiple doses of rifampicin [66,69,70].

Liver cirrhosis is a condition that has been associated with various pathophysiological changes including a reduction in organ blood flows (hepatic and renal), albumin concentration, liver size and changes in abundance of hepatic enzymes that can have a significant impact on exposure of both low and high hepatic clearance drugs [38,71,72]. All of these pathophysiological changes have been incorporated within Simcyp^®^ cirrhosis populations (CP, A-C) [38]. Since the clinical PK data were only available in CP-A population, the developed rifampicin-cirrhosis model was evaluated only in CP-A population [53]. The model predictions in cirrhosis population showed an increase in rifampicin exposure that was consistent with the reported clinical data [73]. In cirrhosis (CP-A) population, the observed and predicted AUC_0–∞_ after administration of 10 mg/kg dose of rifampicin was 68.8 and 86.2 µg/mL·h, respectively [53]. Moreover, in comparison with the healthy adults, the mean observed and predicted CL/F values were lower in cirrhotic patients. The decrease in rifampicin CL/F in liver cirrhosis patients is mainly associated with reduction in liver volume and plasma albumin concentration in these patients [38]. 

The focus of the previously published PBPK models of rifampicin was prediction of DDIs by incorporating data related to CYP3A4 induction after administration of multiple drug doses [25,31]. On the other hand, the present work was focused on understanding rifampicin PK after single-dose administration in healthy and disease populations (tuberculosis and cirrhosis). The presented PBPK models have successfully predicted rifampicin PK after single-dose administration in healthy, tuberculosis and cirrhosis populations as the ratio_(Obs/pred)_ for the PK parameters (AUC_0–∞_, CL/F and C_max_) were within the two-fold error range (Figure 3). 

## 5. Conclusions

The developed drug–disease PBPK models for rifampicin have efficiently predicted rifampicin PK in tuberculosis and cirrhosis populations. The incorporation of plasma albumin concentration changes in tuberculosis population resulted in improved predictions, as the rifampicin-tuberculosis PBPK model has successfully captured the decrease in rifampicin CL/F reported previously in the literature. Furthermore, the addition of pathophysiological changes relevant to cirrhosis in the developed rifampicin-cirrhosis PBPK model resulted in successful prediction of PK parameters. The developed rifampicin-cirrhosis PBPK model was only evaluated with the CP-A population but due to the mechanistic nature of the developed PBPK model it may also be extrapolated to CP-B and C populations, where no relevant clinical PK data is available. The developed rifampicin-cirrhosis PBPK model may have many clinical implications in dose selection for cirrhosis patients. 

## 6. Limitations

For the development and evaluation of rifampicin PBPK models, the systemic concentration vs. time data points were obtained by scanning the figures from the published literature. Although the PK parameters that were derived from the scanned profiles were comparable with the reported values in literature, slight differences cannot be ruled out.

In the presented PBPK models, only CL_iv_ and CL_R_ were used for predicting drug clearance. Although it will be more relevant if we had used individual CYP-enzyme CL, this was not possible due to absence of clear information on individual CYP-enzymes in the literature. 

In order to improve the model predictions, some of the model input parameter values were optimized (B/P, *f*_u_ and P_eff_). The selection of final model input parameters was based on a comparison of observed and predicted profiles after performing sensitivity analysis and manual optimization. 

Due to the presence of clear published information on the decrease in plasma albumin concentration in tuberculosis patients, these pathophysiological changes were most relevant to the ADME of administered drugs in this disease. Therefore, the change in albumin concentration was the only pathophysiological modification that was incorporated into the developed tuberculosis model.

Keeping in view that the primary focus of the presented work was to explore the differences in ADME of rifampicin after application of its single dose in healthy and disease populations, no enzyme induction data was incorporated into the model, and therefore it was not able to account for the auto-induction process associated with multiple-dose application of rifampicin. This inability of the developed models to account for the auto-induction process can be regarded as the main limitation of the presented work.

The disease-related clinical information presented in the study only consisted of albumin and bilirubin values to evaluate rifampicin-cirrhosis PBPK model. Therefore, the calculated CP score was based on these two parameters that lead to categorization of cirrhosis patients into CP-A class. 

## Figures and Tables

**Table 1:** Rifampicin input parameters and characteristics for the developed PBPK model.

Parameters	Reported Values	Model Input Values	Reference
**Physicochemical Properties**			
Molecular weight (g/mol)	822.9	822.9	[34]
Log*P*_o:w_	2.7	2.7	[34]
pK_a_^1^	1.7	1.7	[24]
pK_a_^2^	7.9	7.9	[24]
Compound type	Ampholyte		
**Absorption**			
Model	ADAM		
P_eff,man_ (cm/s)	2.15 × 10^−4^	2.4 × 10^−4 a^	[23]
**Distribution**			
Model	Full PBPK		
Prediction Method 2	Rodger and Rowland method		
B/P ratio	0.52–0.90	0.67 ^a^	[24,25]
*f*_u_	0.15	0.34 ^a^	[24]
V_ss_ (L/kg)	0.33–0.53	0.33	[23,24,25]
**Elimination**			
CL_iv_ (L/h)	7	7	[24]
CL_R_ (L/h)	1.5	1.5	[25]

Log*P*_o:w_: octanol-water partition coefficient, ADAM: advanced dissolution, absorption and metabolism, P_eff_: human jejunum permeability, *f*_u_: fraction of unbound drug in plasma, V_ss_: volume of distribution at steady-state, CL_iv_: intravenous clearance, CL_R_: renal clearance. ^a^ manually optimized values based on visual predictive checks and sensitivity analysis.

**Table 2:** Population data for rifampicin model development in healthy population.

No	Population	No. of Subjects	Dose	Route	Age (Years)	Weight (kg)	Female Proportion	Reference Study *
1	Healthy	2	450 mg	Intravenous infusion	25–60		0	[40]
2	Healthy	6	600 mg	Intravenous infusion	25–60		0	[40]
3	Healthy	12	300 mg	Oral	25–53	48–88	0	[41]
4	Healthy	24	600 mg	Oral	19–45	60–101.4	0	[42]
5	Healthy	18	600 mg	Oral	18–55	>50	0	[43]
6	Healthy	22	600 mg	Oral	18–55	>50	0	[44]
7	Healthy	16	600 mg	Oral	28–59	51–80	0.68	[28]
8	Healthy	18	600 mg	Oral	18–40	Mean: 68.73	0.5	[26]
9	Healthy	66	450 mg	Oral	18–55	>50	0.5	[45]
10	Healthy	61	600 mg	Oral	18–55	>50		[45]
11	Healthy	19	600 mg	Oral	19–29	49–95	0.73	[27]
12	Healthy	8	600 mg	Oral	18–50	Mean: 79.3	0.5	[46]
13	Healthy	6	10 mg/kg	Oral	60–95	44–81	0.33	[47]
14	Healthy	13	450 mg	Oral	18–45		0	[48]
15	Healthy	13	450 mg	Oral	15–59		0	[37]
16	Healthy	30	300 mg	Oral			0.5	[49]
17	Healthy	24	10 mg/kg	Oral	18–65		0.6	[15]

* All the above-mentioned clinical studies were conducted in a fasting state.

**Table 3:** Population data for rifampicin model development in the diseased population.

No.	Population	No. of Subjects	Dose	Route	Age (Years)	Weight (kg)	Female Proportion	Reference Study
1	Tuberculosis	24	10 mg/kg	Oral	18–65		0.6	[15]
2	Tuberculosis	23	600 mg	Oral	18–55	Mean: 47	0.47	[50]
3	Tuberculosis	24	450 mg	Oral	18–55	Mean: 47	0.47	[50]
4	Tuberculosis	18	450 mg	Oral	18–60	47.3	0.61	[51]
5	Tuberculosis	13	450 mg	Oral	15–59		0	[37]
6	Tuberculosis	20	450 mg	Oral	Mean: 40.5	Mean: 42.9	0.4	[52]
7	Liver cirrhosis	7	4 mg/kg	Oral	18–60			[53]
8	Liver cirrhosis	7	6 mg/kg	Oral	18–60			[53]
9	Liver cirrhosis	7	8 mg/kg	Oral	18–60			[53]
10	Liver cirrhosis	7	10 mg/kg	oral	18–60			[53]

**Table 4:** Comparison between observed and predicted pharmacokinetic parameters in healthy, tuberculosis and cirrhosis populations following i.v and oral rifampicin administration.

PK Parameters	Dose	Healthy		Tuberculosis		Liver Cirrhosis	
		Observed	Predicted	Observed	Predicted	Observed	Predicted
**Intravenous Administration**							
**AUC_0–∞_ (µg/mL·h)**	450 mg	52.49	58.16				
	600 mg	73.50	96.38				
**CL (L/h)**	450 mg	7.86	6.23				
	600 mg	8.15	6.22				
**C_max_ (µg/mL)**	450 mg	12.53	9.80				
	600 mg	13.61	13.07				
**Oral Administration**							
**AUC_0–∞_ (µg/mL·h)**	300 mg	29.93	40.19				
	450 mg	42.04	60.20	65.52	64.67		
	600 mg	76.95	81.06	93.40	86.70		
	4 mg/kg					29.6	33.8
	6 mg/kg					70.4	50.7
	8 mg/kg					65.2	67.6
	10 mg/kg	68.85	86.20	66.7	95.3	95.1	84.5
**CL (L/h)**	300 mg	9.98	7.38				
	450 mg	10.77	7.44	7.15	6.92		
	600 mg	8.25	7.44	6.4	0.9		
	4 mg/kg					0.135	0.113
	6 mg/kg					0.085	0.113
	8 mg/kg					0.122	0.113
	10 mg/kg	0.145	0.115	0.14	0.10	0.105	0.113
**C_max_ (µg/mL)**	300 mg	5.37	5.47				
	450 mg	5.88	9.08	8.30	11.45		
	600 mg	9.85	10.30	13.3	15.6		
	4 mg/kg					5.50	5.52
	6 mg/kg					12.10	8.28
	8 mg/kg					11.70	11.04
	10 mg/kg	10.8	10.55	8.50	18.4	17.40	13.80

**Table 5:** Comparison between observed and predicted pharmacokinetic parameters with their observed/predicted ratios, average fold error and root mean square error in healthy, tuberculosis and cirrhosis populations following i.v and oral rifampicin administration.

Parameters	Mean Ratio_obs/pred_ (Range)	AFE	RMSE
**Intravenous Application in Healthy Population**			
AUC_0–∞_ (µg/mL·h)	0.82 (0.76–0.89)	0.78	19.39
CL (L/h)	1.28 (1.26–1.31)	1.27	1.78
C_max_ (µg/mL)	1.16 (1.04–1.27)	1.14	1.96
**Oral Application in Healthy Population**			
AUC_0–∞_ (µg/mL·h)	0.84 (0.51–1.47)	0.80	51.80
CL (L/h)	1.22 (0.65–1.86)	1.14	2.83
C_max_ (µg/mL)	0.88 (0.36–1.67)	0.79	5.95
**Oral Application in Tuberculosis Population**			
AUC_0–∞_ (µg/mL·h)	0.96 (0.69–1.31)	0.93	16.10
CL (L/h)	1.08 (0.76–1.42)	1.02	1.04
C_max_ (µg/mL)	0.69 (0.46–0.85)	0.66	13.82
**Oral Application in Liver Cirrhosis Population**			
AUC_0–∞_ (µg/mL·h)	1.09 (0.87–1.38)	1.30	11.27
CL (L/h)	0.94 (0.72–1.14)	0.98	0.009
C_max_ (µg/mL)	1.19 (0.99–1.46)	1.10	2.67

Ratio_(obs/pred)_: observed/predicted ratio for the pharmacokinetic parameter, AFE: average fold error, RMSE: root mean square error.

## Supplementary Materials

The following are available online at https://www.mdpi.com/1999-4923/11/11/578/s1, Figure S1: Observed and predicted plasma concentration-time profiles of rifampicin in healthy adults (**A**–**C**) and in tuberculosis patients (**D**) after oral administration. Healthy individual after oral administration: (**A**) 300 mg, (**B**) 600 mg, (**C**) 450 mg, (**D**) 450 mg.

## References

[1] Rasool M.F., Khalil F., Läer S. (2015). A Physiologically Based Pharmacokinetic Drug–Disease Model to Predict Carvedilol Exposure in Adult and Paediatric Heart Failure Patients by Incorporating Pathophysiological Changes in Hepatic and Renal Blood Flows. Clin. Pharmacokinet..

[2] Sayama H., Takubo H., Komura H., Kogayu M., Iwaki M. (2014). Application of a physiologically based pharmacokinetic model informed by a top-down approach for the prediction of pharmacokinetics in chronic kidney disease patients. AAPS J..

[3] Tan M.-L., Zhao P., Zhang L., Ho Y.-F., Varma M.V.S., Neuhoff S., Nolin T.D., Galetin A., Huang S.-M. (2019). Use of Physiologically Based Pharmacokinetic Modeling to Evaluate the Effect of Chronic Kidney Disease on the Disposition of Hepatic CYP2C8 and OATP1B Drug Substrates. Clin. Pharmacol. Ther..

[4] Zhao P., Zhang L., Grillo J., Liu Q., Bullock J., Moon Y., Song P., Brar S., Madabushi R., Wu T. (2011). Applications of physiologically based pharmacokinetic (PBPK) modeling and simulation during regulatory review. Clin. Pharmacol. Ther..

[5] Zhuang X., Lu C. (2016). PBPK modeling and simulation in drug research and development. Acta Pharm. Sin. B.

[6] Miller N.A., Reddy M.B., Heikkinen A.T., Lukacova V., Parrott N. (2019). Physiologically Based Pharmacokinetic Modelling for First-In-Human Predictions: An Updated Model Building Strategy Illustrated with Challenging Industry Case Studies. Clin. Pharmacokinet..

[7] Pilari S., Huisinga W. (2010). Lumping of physiologically-based pharmacokinetic models and a mechanistic derivation of classical compartmental models. J. Pharmacokinet. Pharmacodyn..

[8] Shardlow C.E., Generaux G.T., Patel A.H., Tai G., Tran T., Bloomer J.C. (2013). Impact of physiologically based pharmacokinetic modeling and simulation in drug development. Drug Metab. Dispos..

[9] Edginton A.N., Willmann S. (2008). Physiology-based simulations of a pathological condition. Clin. Pharmacokinet..

[10] Khalil F., Läer S. (2011). Physiologically based pharmacokinetic modeling: Methodology, applications, and limitations with a focus on its role in pediatric drug development. Biomed. Res. Int..

[11] Reddy M.B., Clewell H.J., Lave T., Andersen M.E. (2013). Physiologically based pharmacokinetic modeling: A tool for understanding ADMET properties and extrapolating to human. New Insights into Toxicity and Drug Testing.

[12] Zhao P., de LT Vieira M., Grillo J.A., Song P., Wu T.C., Zheng J.H., Arya V., Berglund E.G., Atkinson A.J., Sugiyama Y. (2012). Evaluation of exposure change of nonrenally eliminated drugs in patients with chronic kidney disease using physiologically based pharmacokinetic modeling and simulation. J. Clin. Pharmacol..

[13] Verscheijden L.F., Koenderink J.B., de Wildt S.N., Russel F.G. (2019). Development of a physiologically-based pharmacokinetic pediatric brain model for prediction of cerebrospinal fluid drug concentrations and the influence of meningitis. PLoS Comput. Biol..

[14] Marsousi N., Desmeules J.A., Rudaz S., Daali Y. (2017). Usefulness of PBPK modeling in incorporation of clinical conditions in personalized medicine. J. Pharm. Sci..

[15] Medellín-Garibay S., Cortez-Espinosa N., Milán-Segovia R., Magaña-Aquino M., Vargas-Morales J., González-Amaro R., Portales-Pérez D., Romano-Moreno S. (2015). Clinical pharmacokinetics of rifampicin in patients with tuberculosis and type 2 diabetes mellitus: Association with biochemical and immunological parameters. Antimicrob. Agents Chemother..

[16] Denholm J.T., McBryde E.S. (2010). The use of anti-tuberculosis therapy for latent TB infection. Infect. Drug Resist..

[17] Ramakrishnan K., Shenbagarathai R., Kavitha K., Uma A., Balasubramaniam R., Thirumalaikolundu Subramanian P. (2008). Serum zinc and albumin levels in pulmonary tuberculosis patients with and without HIV. Jpn. J. Infect. Dis..

[18] Sudfeld C.R., Isanaka S., Aboud S., Mugusi F.M., Wang M., Chalamilla G.E., Fawzi W.W. (2013). Association of serum albumin concentration with mortality, morbidity, CD4 T-cell reconstitution among tanzanians initiating antiretroviral therapy. J. Infect. Dis..

[19] Rowland M. (1984). Protein binding and drug clearance. Clin. Pharmacokinet..

[20] Glaeser H., Drescher S., Eichelbaum M., Fromm M. (2005). Influence of rifampicin on the expression and function of human intestinal cytochrome P450 enzymes. Br. J. Clin. Pharmacol..

[21] Agrawal S., Panchagnula R. (2005). Implication of biopharmaceutics and pharmacokinetics of rifampicin in variable bioavailability from solid oral dosage forms. Biopharm. Drug Dispos..

[22] Svensson R.J., Aarnoutse R.E., Diacon A.H., Dawson R., Gillespie S.H., Boeree M.J., Simonsson U.S. (2018). A population pharmacokinetic model incorporating saturable pharmacokinetics and autoinduction for high rifampicin doses. Clin. Pharmacol. Ther..

[23] Gu H., Dutreix C., Rebello S., Ouatas T., Wang L., Chun D.Y., Einolf H.J., He H. (2018). Simultaneous Physiologically Based Pharmacokinetic (PBPK) Modeling of Parent and Active Metabolites to Investigate Complex CYP3A4 Drug-Drug Interaction Potential: A Case Example of Midostaurin. Drug Metab. Dispos..

[24] Varma M.V., Lai Y., Feng B., Litchfield J., Goosen T.C., Bergman A. (2012). Physiologically based modeling of pravastatin transporter-mediated hepatobiliary disposition and drug-drug interactions. Pharm. Res..

[25] Baneyx G., Parrott N., Meille C., Iliadis A., Lavé T. (2014). Physiologically based pharmacokinetic modeling of CYP3A4 induction by rifampicin in human: Influence of time between substrate and inducer administration. Eur. J. Pharm. Sci..

[26] Milan-Segovia R., Dominguez-Ramirez A., Jung-Cook H., Magana-Aquino M., Romero-Mendez M., Medellin-Garibay S., Vigna-Perez M., Romano-Moreno S. (2010). Relative bioavailability of rifampicin in a three-drug fixed-dose combination formulation. Int. J. Tuberc. Lung Dis..

[27] Pähkla R., Lambert J., Ansko P., Winstanley P., Davies P., Kiivet R.A. (1999). Comparative bioavailability of three different preparations of rifampicin. J. Clin. Pharm. Ther..

[28] Zwolska Z., Augustynowicz-Kopec E., Niemirowska-Mikulska H. (2002). The pharmacokinetic factors and bioavailability of rifampicin, isoniazid and pyrazinamid fixed in one dose capsule. Acta Pol. Pharm..

[29] Khadka P., Dummer J., Hill P.C., Das S.C. (2018). Considerations in preparing for clinical studies of inhaled rifampicin to enhance tuberculosis treatment. Int. J. Pharm..

[30] Loos U., Musch E., Jensen J., Mikus G., Schwabe H., Eichelbaum M. (1985). Pharmacokinetics of oral and intravenous rifampicin during chronic administration. Klin. Wochenschr..

[31] Asaumi R., Toshimoto K., Tobe Y., Hashizume K., Nunoya K.i., Imawaka H., Lee W., Sugiyama Y. (2018). Comprehensive PBPK Model of Rifampicin for Quantitative Prediction of Complex Drug-Drug Interactions: CYP3A/2C9 Induction and OATP Inhibition Effects. CPT Pharmacomet. Syst. Pharmacol..

[32] Jamei M., Marciniak S., Feng K., Barnett A., Tucker G., Rostami-Hodjegan A. (2009). The Simcyp^®^ population-based ADME simulator. Expert Opin. Drug Metab. Toxicol..

[33] Khalil F., Laer S. (2014). Physiologically based pharmacokinetic models in the prediction of oral drug exposure over the entire pediatric age range—Sotalol as a model drug. AAPS J..

[34] PubChem Rifmpicin (Compound Summary). https://pubchem.ncbi.nlm.nih.gov/compound/Rifampicin.

[35] Jamei M., Turner D., Yang J., Neuhoff S., Polak S., Rostami-Hodjegan A., Tucker G. (2009). Population-based mechanistic prediction of oral drug absorption. AAPS J..

[36] Rodgers T., Rowland M. (2007). Mechanistic approaches to volume of distribution predictions: Understanding the processes. Pharm. Res..

[37] Rafiq S., Iqbal T., Jamil A., Khan F.H. (2010). Pharmacokinetic studies of rifampicin in healthy volunteers and tuberculosis patients. Int. J. Agric. Biol..

[38] Johnson T.N., Boussery K., Rowland-Yeo K., Tucker G.T., Rostami-Hodjegan A. (2010). A semi-mechanistic model to predict the effects of liver cirrhosis on drug clearance. Clin. Pharmacokinet..

[39] GetData Graph Digitizer. http://getdata-graph-digitizer.com/.

[40] Nitti V., Virgilio R., Patricolo M., Iuliano A. (1977). Pharmacokinetic study of intravenous rifampicin. Chemotherapy.

[41] Chouchane N., Barre J., Toumi A., Tillement J., Benakis A. (1995). Bioequivalence study of two pharmaceutical forms of rifampicin capsules in man. Eur. J. Drug Metab. Pharmacokinet..

[42] Peloquin C.A., Jaresko G.S., Yong C.-L., Keung A., Bulpitt A.E., Jelliffe R.W. (1997). Population pharmacokinetic modeling of isoniazid, rifampin, and pyrazinamide. Antimicrob. Agents Chemother..

[43] Xu J., Jin H., Zhu H., Zheng M., Wang B., Liu C., Chen M., Zhou L., Zhao W., Fu L. (2013). Oral bioavailability of rifampicin, isoniazid, ethambutol, and pyrazinamide in a 4-drug fixed-dose combination compared with the separate formulations in healthy Chinese male volunteers. Clin. Ther..

[44] Agrawal S., Singh I., Kaur K.J., Bhade S.R., Kaul C.L., Panchagnula R. (2004). Comparative bioavailability of rifampicin, isoniazid and pyrazinamide from a four drug fixed dose combination with separate formulations at the same dose levels. Int. J. Pharm..

[45] Agrawal S., Singh I., Kaur K.J., Bhade S., Kaul C.L., Panchagnula R. (2004). Bioequivalence trials of rifampicin containing formulations: Extrinsic and intrinsic factors in the absorption of rifampicin. Pharmacol. Res..

[46] Peloquin C.A., Namdar R., Singleton M.D., Nix D.E. (1999). Pharmacokinetics of rifampin under fasting conditions, with food, and with antacids. Chest.

[47] Advenier C., Gobert C., Houin G., Bidet D., Richelet S., Tillement J. (1983). Pharmacokinetic studies of rifampicin in the elderly. Ther. Drug Monit..

[48] Agrawal S., Kaur K.J., Singh I., Bhade S.R., Kaul C.L., Panchagnula R. (2002). Assessment of bioequivalence of rifampicin, isoniazid and pyrazinamide in a four drug fixed dose combination with separate formulations at the same dose levels. Int. J. Pharm..

[49] Marchidanu D., Raducanu N., Miron D.S., Radulescu F., Anuta V., Mircioiu I., Prasacu I. (2013). Comparative pharmacokinetics of rifampicin and 25-desacetyl rifampicin in healthy volunteers after single oral dose administration. FARMACIA.

[50] Ruslami R., Nijland H.M., Alisjahbana B., Parwati I., van Crevel R., Aarnoutse R.E. (2007). Pharmacokinetics and tolerability of a higher rifampin dose versus the standard dose in pulmonary tuberculosis patients. Antimicrob. Agents Chemother..

[51] Ruslami R., Nijland H.M., Adhiarta I.G.N., Kariadi S.H., Alisjahbana B., Aarnoutse R.E., van Crevel R. (2010). Pharmacokinetics of antituberculosis drugs in pulmonary tuberculosis patients with type 2 diabetes. Antimicrob. Agents Chemother..

[52] Saktiawati A.M., Sturkenboom M.G., Stienstra Y., Subronto Y.W., Kosterink J.G., van der Werf T.S., Alffenaar J.-W.C. (2015). Impact of food on the pharmacokinetics of first-line anti-TB drugs in treatment-naive TB patients: A randomized cross-over trial. J. Antimicrob. Chemother..

[53] Curci G., Claar E., Bergamini N., Ninni A., Claar G., Ascione A., Nitti V. (1973). Studies on blood serum levels of rifampicin in patients with normal and impaired liver function. Chemother..

[54] Marsousi N., Daali Y., Rudaz S., Almond L., Humphries H., Desmeules J., Samer C.F. (2014). Prediction of Metabolic Interactions with Oxycodone via CYP2D6 and CYP3A Inhibition Using a Physiologically Based Pharmacokinetic Model. CPT Pharmacomet. Syst. Pharmacol..

[55] Jiang X.L., Zhao P., Barrett J.S., Lesko L.J., Schmidt S. (2013). Application of physiologically based pharmacokinetic modeling to predict acetaminophen metabolism and pharmacokinetics in children. CPT Pharmacomet. Syst Pharm..

[56] Jamei M., Bajot F., Neuhoff S., Barter Z., Yang J., Rostami-Hodjegan A., Rowland-Yeo K. (2014). A mechanistic framework for in vitro-in vivo extrapolation of liver membrane transporters: Prediction of drug-drug interaction between rosuvastatin and cyclosporine. Clin. Pharm..

[57] Chen Y., Cabalu T.D., Callegari E., Einolf H., Liu L., Parrott N., Peters S.A., Schuck E., Sharma P., Tracey H. (2019). Recommendations for the Design of Clinical Drug-Drug Interaction Studies with Itraconazole using a Mechanistic PBPK Model. CPT Pharmacomet. Syst. Pharmacol..

[58] Chen Y., Ma F., Lu T., Budha N., Jin J.Y., Kenny J.R., Wong H., Hop C.E.C.A., Mao J. (2016). Development of a Physiologically Based Pharmacokinetic Model for Itraconazole Pharmacokinetics and Drug–Drug Interaction Prediction. Clin. Pharmacokinet..

[59] Heimbach T., Lin W., Hourcade-Potelleret F., Tian X., Combes F.P., Horvath N., He H. (2019). Physiologically Based Pharmacokinetic Modeling to Supplement Nilotinib Pharmacokinetics and Confirm Dose Selection in Pediatric Patients. J. Pharm. Sci..

[60] Zhang Y., Huo M., Zhou J., Xie S. (2010). PKSolver: An add-in program for pharmacokinetic and pharmacodynamic data analysis in Microsoft Excel. Comput. Methods Programs Biomed..

[61] De Buck S.S., Sinha V.K., Fenu L.A., Nijsen M.J., Mackie C.E., Gilissen R.A. (2007). Prediction of human pharmacokinetics using physiologically based modeling: A retrospective analysis of 26 clinically tested drugs. Drug Metab. Dispos..

[62] Li G.-F., Wang K., Chen R., Zhao H.-R., Yang J., Zheng Q.-S. (2012). Simulation of the pharmacokinetics of bisoprolol in healthy adults and patients with impaired renal function using whole-body physiologically based pharmacokinetic modeling. Acta Pharmacol. Sin..

[63] Verscheijden L., Koenderink J., Allegaert K., de Wildt S., Russel F. (2019). O19 Development of a paediatric brain PBPK model in children with and without meningitis. Arch. Dis. Child..

[64] Shaheen A., Najmi M.H., Saeed W., Farooqi Z.-U.-R. (2012). Pharmacokinetics of standard dose regimens of rifampicin in patients with pulmonary tuberculosis in Pakistan. Scand. J. Infect. Dis..

[65] Benet L.Z., Hoener B.A. (2002). Changes in plasma protein binding have little clinical relevance. Clin. Pharmacol. Ther..

[66] Kenny M., Strates B. (1981). Metabolism and pharmacokinetics of the antibiotic rifampin. Drug Metab. Rev..

[67] Loos U., Musch E., Jensen J., Schwabe H., Eichelbaum M. (1987). Influence of the enzyme induction by rifampicin on its presystemic metabolism. Pharmacol. Ther..

[68] CYP2C9 C.C., CYP2D6 C.A. (2007). The effect of cytochrome P450 metabolism on drug response, interactions, and adverse effects. Am. Fam. Phys..

[69] Polasa K., Murthy K., Krishnaswamy K. (1984). Rifampicin kinetics in undernutrition. Br. J. Clin. Pharmacol..

[70] Um S., Lee S., Kwon S., Yoon H.I., Park K.U., Song J., Lee C.T., Lee J. (2007). Low serum concentrations of anti-tuberculosis drugs and determinants of their serum levels. Int. J. Tuberc. Lung Dis..

[71] Westphal J.-F., Brogard J.-M. (1997). Drug administration in chronic liver disease. Drug Saf..

[72] Verbeeck R.K. (2008). Pharmacokinetics and dosage adjustment in patients with hepatic dysfunction. Eur. J. Clin. Pharmacol..

[73] Capelle P., Dhumeaux D., Mora M., Feldmann G., Berthelot P. (1972). Effect of rifampicin on liver function in man. Gut.

