# Supplementary material for: Development and Evaluation of Physiologically Based Pharmacokinetic Drug–Disease Models for Predicting Rifampicin Exposure in Tuberculosis and Cirrhosis Populations
Source: Pharmaceutics. 2019 Nov 5;11(11):578. doi: 10.3390/pharmaceutics11110578 (PMC6921057; doi:10.3390/pharmaceutics11110578)
Supplement: Supplementary file 1 [file pharmaceutics-11-00578-s001.pdf]

pharmaceutics

MDPI

Supplementary Materials: Development and Evaluation of Physiologically Based Pharmacokinetic Drug–Disease Models for Predicting Rifampicin Exposure in Tuberculosis and Cirrhosis Populations

Muhammad F. Rasool, Sundus Khalid, Abdul Majeed, Hamid Saeed, Imran, Mohamed Mohany, Salim S. Al-Rejaie and Faleh Alqahtani

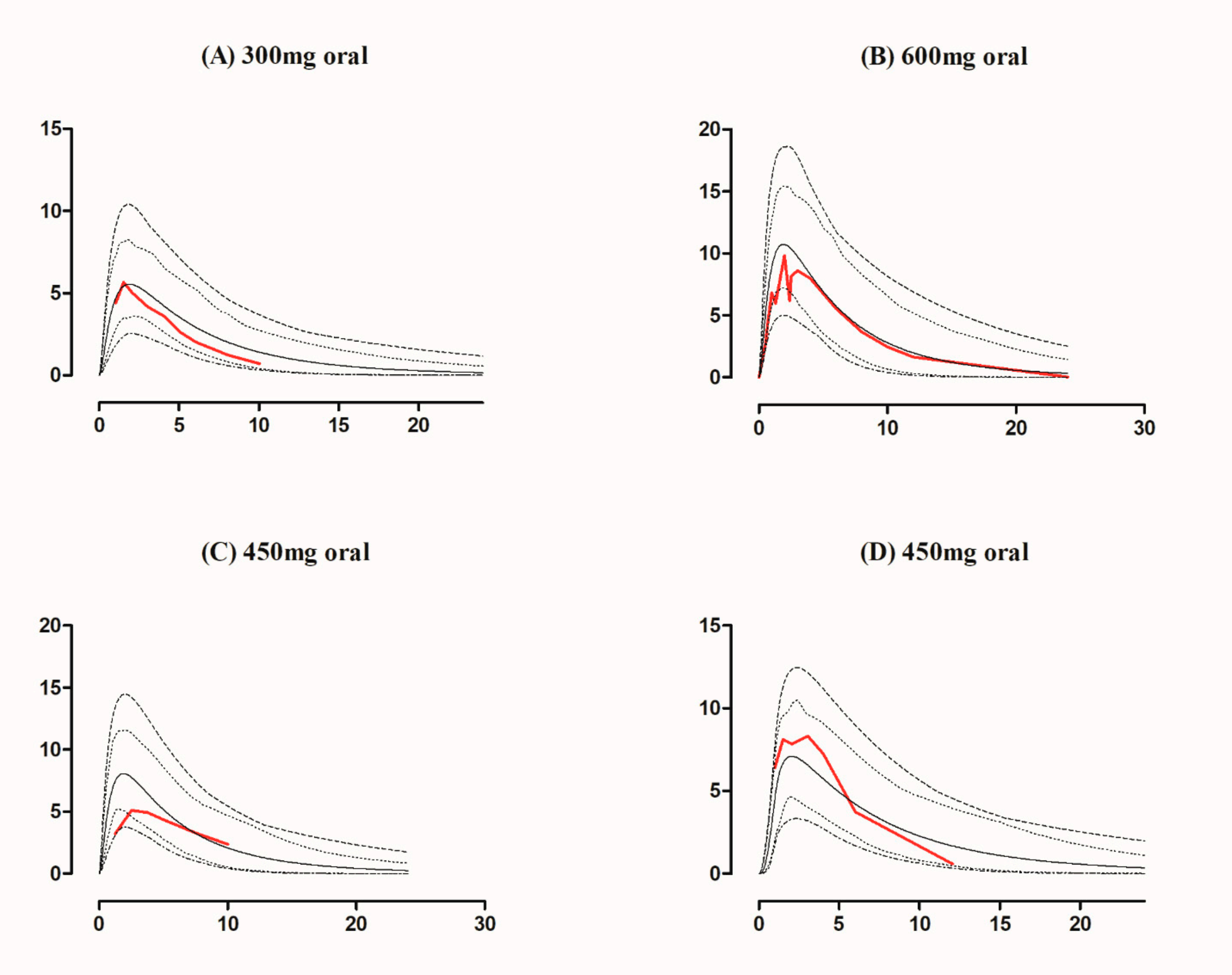

Figure S1. observed and predicted plasma concentration-time profiles of rifampicin in healthy adults (**A**, **B**, **C**) and in tuberculosis patients (**D**) after oral administration. Healthy individual after oral administration: (**A**) 300 mg [1,2], (**B**) 600 mg [3–10], (**C**) 450 mg [8,11,12], (**D**) 450 mg [12–15]. The median observed data is shown as solid red line. The predicted results are shown as mean (solid line), maximum value and minimum value (dashed line) and the 5th–95th percentiles (dotted line).

References

1. Chouchane, N.; Barre, J.; Toumi, A.; Tillement, J.; Benakis, A. Bioequivalence study of two pharmaceutical forms of rifampicin capsules in man. *Eur. J. Drug Metab. Pharmacokinet.* **1995**, *20*, 315–320.
2. Marchidanu, D.; Raducanu, N.; Miron, D.S.; Radulescu, F.; Anuta, V.; Mircioiu, I.; Prasacu, I. Comparative pharmacokinetics of rifampicin and 25-desacetyl rifampicin in healthy volunteers after single oral dose administration. *FARMACIA* **2013**, *61*, 398–410.

3. Peloquin, C.A.; Jaresko, G.S.; Yong, C.-L.; Keung, A.; Bulpitt, A.E.; Jelliffe, R.W. Population pharmacokinetic modeling of isoniazid, rifampin, and pyrazinamide. *Antimicrob. Agents Chemother.* **1997**, *41*, 2670–2679.
4. Xu, J.; Jin, H.; Zhu, H.; Zheng, M.; Wang, B.; Liu, C.; Chen, M.; Zhou, L.; Zhao, W.; Fu, L. Oral bioavailability of rifampicin, isoniazid, ethambutol, and pyrazinamide in a 4-drug fixed-dose combination compared with the separate formulations in healthy Chinese male volunteers. *Clin. Ther.* **2013**, *35*, 161–168.
5. Agrawal, S.; Singh, I.; Kaur, K.J.; Bhade, S.R.; Kaul, C.L.; Panchagnula, R. Comparative bioavailability of rifampicin, isoniazid and pyrazinamide from a four drug fixed dose combination with separate formulations at the same dose levels. *Int. J. Pharm.* **2004**, *276*, 41–49.
6. Zwolska, Z.; Augustynowicz-Kopec, E.; Niemirowska-Mikulska, H. The pharmacokinetic factors and bioavailability of rifampicin, isoniazid and pyrazinamid fixed in one dose capsule. *Acta Pol. Pharm.* **2002**, *59*, 448–451.
7. Milan-Segovia, R.; Dominguez-Ramirez, A.; Jung-Cook, H.; Magana-Aquino, M.; Romero-Mendez, M.; Medellin-Garibay, S.; Vigna-Perez, M.; Romano-Moreno, S. Relative bioavailability of rifampicin in a three-drug fixed-dose combination formulation. *Int. J. Tuberc. Lung Dis.* **2010**, *14*, 1454–1460.
8. 8. Agrawal, S.; Singh, I.; Kaur, K.J.; Bhade, S.; Kaul, C.L.; Panchagnula, R. Bioequivalence trials of rifampicin containing formulations: Extrinsic and intrinsic factors in the absorption of rifampicin. *Pharmacol. Res.* **2004**, *50*, 317–327.
9. Pähkla, R.; Lambert, J.; Ansko, P.; Winstanley, P.; Davies, P.; Kiivet, R.A. Comparative bioavailability of three different preparations of rifampicin. *J. Clin. Pharm. Ther.* **1999**, *24*, 219–225.
10. Peloquin, C.A.; Namdar, R.; Singleton, M.D.; Nix, D.E. Pharmacokinetics of rifampin under fasting conditions, with food, and with antacids. *Chest* **1999**, *115*, 12–18.
11. Agrawal, S.; Kaur, K.J.; Singh, I.; Bhade, S.R.; Kaul, C.L.; Panchagnula, R. Assessment of bioequivalence of rifampicin, isoniazid and pyrazinamide in a four drug fixed dose combination with separate formulations at the same dose levels. *Int. J. Pharm.* **2002**, *233*, 169–177.
12. Rafiq, S.; Iqbal, T.; Jamil, A.; Khan, F.H. Pharmacokinetic studies of rifampicin in healthy volunteers and tuberculosis patients. *Int. J. Agric. Biol.* **2010**, *12*, 391–395.
13. Ruslami, R.; Nijland, H.M.; Alisjahbana, B.; Parwati, I.; van Crevel, R.; Aarnoutse, R.E. Pharmacokinetics and tolerability of a higher rifampin dose versus the standard dose in pulmonary tuberculosis patients. *Antimicrob. Agents Chemother.* **2007**, *51*, 2546–2551.
14. Ruslami, R.; Nijland, H.M.; Adhiarta, I.G.N.; Kariadi, S.H.; Alisjahbana, B.; Aarnoutse, R.E.; van Crevel, R. Pharmacokinetics of antituberculosis drugs in pulmonary tuberculosis patients with type 2 diabetes. *Antimicrob. Agents Chemother.* **2010**, *54*, 1068–1074.
15. Saktiawati, A.M.; Sturkenboom, M.G.; Stienstra, Y.; Subronto, Y.W.; Kosterink, J.G.; van der Werf, T.S.; Alffenaar, J.-W.C. Impact of food on the pharmacokinetics of first-line anti-TB drugs in treatment-naive TB patients: A randomized cross-over trial. *J. Antimicrob. Chemother.* **2015**, *71*, 703–710.
